# Physiological Significance of Ion Transporters and Channels in the Stomach and Pathophysiological Relevance in Gastric Cancer

**DOI:** 10.1155/2020/2869138

**Published:** 2020-02-12

**Authors:** Dumin Yuan, Zhiyuan Ma, Biguang Tuo, Taolang Li, Xuemei Liu

**Affiliations:** ^1^Department of Gastroenterology, Affiliated Hospital of Zunyi Medical University, Zunyi, Guizhou Province, China; ^2^Digestive Disease Institute of Guizhou Province, Zunyi, Guizhou Province, China; ^3^Department of Thyroid and Breast Surgery, Affiliated Hospital of Zunyi Medical University, Zunyi, Guizhou Province, China

## Abstract

Gastric cancer (GC) is a highly invasive and fatal malignant disease that accounts for 5.7% of new global cancer cases and is the third leading cause of cancer-related death. Acid/base homeostasis is critical for organisms because protein and enzyme function, cellular structure, and plasma membrane permeability change with pH. Various ion transporters are expressed in normal gastric mucosal epithelial cells and regulate gastric acid secretion, ion transport, and fluid absorption, thereby stabilizing the differentiation and homeostasis of gastric mucosal epithelial cells. Ion transporter dysfunction results in disordered ion transport, mucosa barrier dysfunction, and acid/base disturbances, causing gastric acid-related diseases such as chronic atrophic gastritis (CAG) and GC. This review summarizes the physiological functions of multiple ion transporters and channels in the stomach, including Cl^−^ channels, Cl^−^/HCO_3_^−^ exchangers, sodium/hydrogen exchangers (NHEs), and potassium (K^+^) channels, and their pathophysiological relevance in GC.

## 1. Introduction

**Figure 1:**
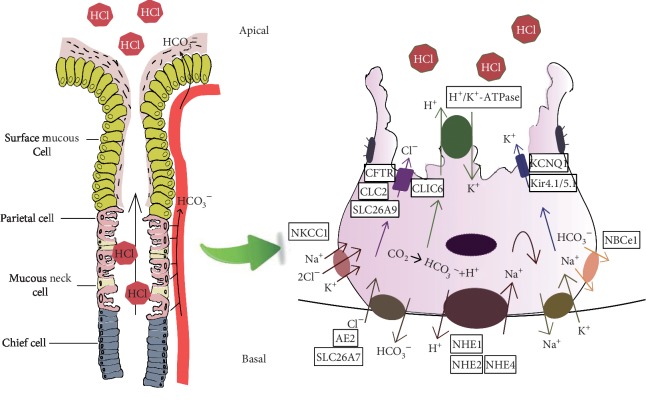
Ion transporters and channels related to acid/base homeostasis and their localization in parietal cells. (a) Normal structure of the oxyntic gland and acid/base homeostasis in the stomach. (b) Multiple ion transporters and channels are located in the parietal cell and are involved in the regulation of the HCl output; these include Cl^−^ channels: CFTR, CLC2, CLIC6, and SLC26A9; Cl^−^/HCO_3_^−^ exchangers: SLC26A7 and AE2; sodium/hydrogen exchangers (NHEs): NHE1, NHE2, and NHE4; potassium (K^+^) channels: KCNQ1 and Kir4.1/5.1; the Na^+^-K^+^-2Cl^−^ cotransporter: NKCC1; the Na^+^-HCO_3_^−^ cotransporter: NBCe1; and H^+^/K^+^-ATPase. The arrows indicate the direction of ion transport.

**Figure 2:**
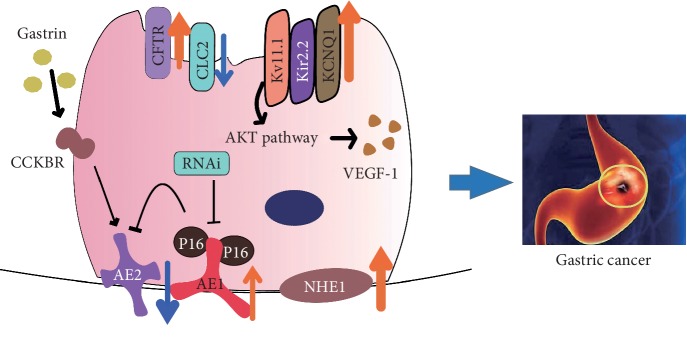
Dysfunction of ion transporters and channels in parietal cells resulting in GC onset. Upregulation of CFTR, AE1, NHE1, Kv11.1, Kir2.2, and KCNQ1, downregulation of AE2, and other interactions in parietal cells result in disorder of different signaling pathways, thereby inducing the GC onset (black arrow indicates activation; black “T” indicates inhibition; orange arrow indicates upregulation; and blue arrow indicates downregulation).

Gastric cancer (GC) is one of the most aggressive types of cancer and a major health threat and contributor to cancer-related death worldwide [1]. Therefore, clarification of its etiology pathogenesis is important for the identification of effective therapeutic targets for early diagnosis and prevention. According to the classic “Correa sequence,” GC develops via a stepwise progression through a sequence of histopathologic changes [2, 3], including chronic atrophic gastritis (CAG), metaplasia, dysplasia, and eventually neoplasia [2]. Parietal cell loss is the critical and initial step necessary for GC development [4–12]. Too little acid secretion promotes excessive bacterial growth in the gastrointestinal (GI) tract, which triggers the upregulation of related inflammatory factors and leads to intragastric infection and CAG, eventually progressing to GC [7, 8]. Additionally, loss of parietal cells results in deficiencies of a series of important mucosal growth factors, including transforming growth factor alpha (TGF-*α*), amphiregulin, heparin-binding epidermal growth factor (HB-EGF), and sonic hedgehog, thereby causing the transduction of chief cells into spasmolytic polypeptide-expressing metaplasia (SPEM) [9, 10], an important precancerous lesion of GC [11, 12]. In the normal stomach, acid secretion by parietal cells requires a functional H^+^/K^+^-ATPase, apical Cl^−^ secretion, and K^+^ recycling, as well as basolateral HCO_3_^−^ and Cl^−^ exchange (Figure 1(b)). Parietal cells actively pump out H^+^ against a strong concentration gradient by the ATP-driven exchange of one H^+^ for one K^+^ via the enzyme H^+^/K^+^-ATPase [13]. Cl^−^ is extruded concurrently with H^+^ across the luminal membrane, and these ions combine to form HCl; this process relies on the cooperation of various ion transporters and channels in parietal cells of oxyntic mucosa [14] (Figure 1; Table 1). Moreover, gastric surface cells provide the first line of defense against acidic chambers and establish an alkaline environment near the apical cell surface to prevent acid damage to intestinal cells, the so-called “epithelial-bicarbonate barrier,” which is an important structure for gastric mucosal protection. During this process, some ion transporters, channels, and enzymes are involved in supporting gastric bicarbonate secretion [15–18]. Taken together, these observations reveal that ion transporters and channels play an important role in regulating ion transport, mucus barrier function, signaling pathways, and acid/base homeostasis in the stomach. A previous study implicated dysfunction of the “ion transport mechanism” (ITM), which is involved in regulating GC cell proliferation, apoptosis, differentiation, and progression via different signaling pathways, in carcinogenesis [19–22]. Therefore, this review summarizes the physiological functions of different ion transporters and channels, including Cl^−^ channels, Cl^−^/HCO_3_^−^ exchangers, sodium/hydrogen exchangers (NHEs), and potassium (K^+^) channels, in parietal cells (Figure 1(b); Table 1), and their pathophysiological relevance in GC (Figure 2; Table 1) to provide new research directions to understand the molecular mechanism of this malignant disease.

## 2. Physiological Characteristics of Cl^−^ Channels in the Stomach and Pathophysiological Relevance in GC

### 2.1. Role of CFTR in GC

#### 2.1.1. Physiological Function of CFTR in the Stomach

CFTR is a member of the membrane transporter ATP-binding cassette (ABC) family that comprises 48 members in humans subdivided into 7 subfamilies (ABCA–ABCG) [39]. Most ABC proteins act as active ATP-dependent transporters that couple ATP binding and hydrolysis to unidirectional transport across the matrix [40, 41]. Among human ABC proteins, CFTR is considered unique because it has no active transport function but rather acts as a phosphorylation-regulated ATP-gated anion channel [42]; it has a physiological role in transporting salt and water in epithelial cells [43] and mainly mediates the passive transport of Cl^−^ and HCO_3_^−^ [44–46]. CFTR is regulated by phosphorylation [47–49] and ATP binding and hydrolysis [50–52]. It is widely expressed throughout the body but is mainly localized on the apical (cavity) membrane of ductal and ductal epithelial cells, where it is involved in transepithelial fluid and electrolyte transport and intracellular pH (pHi) regulation [53, 54].

CFTR is highly expressed in the apical lining of crypt epithelial cells [55, 56] and functions as an important regulator of intestinal homeostasis [57]. Mutations in the *CFTR* gene affect chloride channel function, resulting in the dysregulation of epithelial fluids and salt transport in many organs, including the lung, stomach, and intestinal digestive system, ultimately causing cystic fibrosis (CF) [58]. In the stomach, despite the detection of a low CFTR expression level [59, 60], the CFTR channel inhibitor CFTR-inh172 abolishes acid secretion in mice [61–63]. A potential mechanism for this effect is that CFTR may act as the ATP-binding cassette transporter associated with Kir2.1 in parietal cells to modulate H^+^-K^+^-ATPase-mediated secretagogue-induced acid secretion [63, 64].

#### 2.1.2. Pathophysiological Role of CFTR in GC

Eberle et al. showed that brush cells in the “gastric groove” may not be the source of the alkaline solution but rather promote bicarbonate secretion and protect the gastric mucosa from gastric acid through the paracrine production of prostaglandins that activate nearby CFTR-positive cells [65]. El-Zimaity et al. investigated this issue in the stomach upon the loss of parietal cells due to *Helicobacter pylori* infection, which results in an inflammatory response and SPEM, another significant precancerous lesion of GC [11, 12]. CFTR mRNA expression is upregulated under these conditions [23], suggesting that CFTR may promote GC by affecting SPEM lesions. However, later experiments showed that CFTR is closely related to classical tumor biomarker carbohydrate antigen 199 (CA199) in GC, and CFTR expression increases with age and is associated with the clinical stage of GC. Therefore, serum CFTR has a wide range of applications for GC detection [24]. Additionally, some studies have demonstrated that CFTR activity inhibition suppresses the division of the human GC cell line MKN45 [66]. Therefore, CFTR may be a new target for the prevention and treatment of GC.

### 2.2. Physiological Characteristics of CLC-2 and GC

#### 2.2.1. Physiological Function of CLC-2 in the Stomach

CLC-2 is a widely expressed Cl^−^ channel that can be activated by hyperpolarization, extracellular (luminal) acidic pH, and fatty acid-activated omeprazole in rabbits and humans but not by mouse protein kinase A [67–76]. Moreover, CLC-2 can be activated by protein kinase C [77]. Secretagogue stimulation results in a major rapid morphological transformation in parietal cells, which is essential for maximal acid secretion; in this process, cytoplasmic tubulovesicles containing H^+^/K^+^-ATPase (and perhaps Cl^−^ and K^+^ channels/transporters) fuse with the apical membrane to form a greatly expanded secretory canaliculus with increased elongated microvilli that are recycled during the resting stage [13, 78, 79]. CLC-2 localized to gastric parietal cells in isolated rabbit gastric glands showed similar localization to H^+^/K^+^-ATPase and was important for gastric parietal cell acid secretion [80]. CLC-2 was detected in porcine gastric mucosa, and the CLC-2 agonist SPI-8811 was reported to rescue gastric mucosal barrier function and ameliorate acid-induced gastric injury [81]. However, other studies concluded that CLC-2 is not involved in gastric acid secretion [82]. Thus, further research is required to elucidate the function of CLC-2 in the stomach.

#### 2.2.2. Pathophysiological Role of CLC-2 in GC

Initially, some groups investigated whether genetic ablation of CLC-2 affects the gastric mucosa with a focus on parietal cell abundance, H^+^/K^+^-ATPase expression, morphology, and acid secretion using CLC-2^+/+^ and CLC-2^−/−^ mice. The researchers reported that CLC-2 colocalizes with H^+^/K^+^-ATPase in gastric parietal cells. Deletion of CLC-2 resulted in a series of morphological changes in the gastric mucosa, as observed by electron microscopy: gastric gland dilation, reduced height of the gastric gland region, parietal cell loss, reduced parietal cell H^+^/K^+^-ATPase expression, and tubulovesicles without expanded canaliculi [25]. Thus, CLC-2 influences gastric acid secretion to a certain extent. The morphological changes in the gastric mucosa were exactly the same as the precancerous changes mentioned above. Although no relevant studies have examined the relationship between CLC-2 and GC directly, CLC-2 may play a crucial role in the maintenance of gastric mucosal homeostasis and, thus, may play a role in the development of CAG and GC.

## 3. Physiological Characteristics of Cl^−^/HCO_3_^−^ Exchangers in the Stomach and Pathophysiological Relevance in GC

### 3.1. Physiological Function of the SLC4 Family in the Stomach

The human SLC4 family consists of 10 genes encoding secondary transporters for bicarbonate and/or carbonate [83, 84]. The SLC4 family is divided into three major branches: electrically neutral Na^+^-independent Cl^−^/HCO_3_^−^ antiporters, including SLC4A1 (AE1), SLC4A2 (AE2), and SLC4A3 (AE3); Na^+^-dependent SLC4 HCO_3_^−^ transporters, including electricity-producing SLC4A4 (NBCe1) and SLC4A5 (NBCe2), electrically neutral Na^+^/HCO_3_^−^ cotransporters SLC4A7 (NBCn1) and SLC4A10 (NBCn2), and a Na^+^-2HCO_3_^−^/Cl^−^ exchanger; and a branch with one unusual member (SLC4A9) that has been described as being capable of most of the above actions [83, 85–87]. Here, we discuss the most significant Cl^−^/HCO_3_^−^ antiporters, AE1 and AE2, in the stomach.

SLC4A1 (AE1) is the major glycoprotein of the erythrocyte membrane, with more than 1 million copies per cell [88–90]. This protein is an important member of the solute carrier SLC4 series of bicarbonate transporters [91]. The human AE1 protein is not expressed in the normal stomach [92]. The *AE2* gene (also known as SLC4A2) encodes a Na^+^-independent, electroneutral Cl^−^/HCO_3_^−^ exchanger [93] that localizes to the membrane and is relevant for pHi regulation and bicarbonate secretion in several cell types. AE2 appears to primarily increase intracellular acidification since its activity is responsive to increased pHi. In addition, AE2 regulates the intracellular chloride concentration, bicarbonate metabolism, and cell volume in a wide variety of cell types [94–96]. AE2-null mice were reported to have severe defects in acid secretion; however, morphological studies of these mice revealed abnormal gastric morphology, and most mice died around the time of weaning, making the data difficult to interpret [97]. As Cl^−^/HCO_3_^−^ exchangers, AE1 and AE2 play important roles in maintaining gastric acid/base homeostasis and secreting gastric acid.

### 3.2. Pathophysiological Role of the SLC4 Family in GC

Wang et al. analyzed 182 cases of advanced GC and found that AE1 expression in the cytoplasm of GC cells increased in the late stage of GC. The C-terminal 112 residues of AE1 interact with the tumor suppressor p16 [98], indicating that AE1 is an indicator of malignant GC [33]. Moreover, the cytoplasmic AE1/p16 complex enhances the stability of both proteins and plays a key role in GC progression; thus, this complex is associated with a decreased patient survival time [33–35]. Recent studies have reported that AE2 is downregulated in GC cells, and this downregulation correlates with carcinogenesis and is blocked by gastrin [36]. Recently, researchers suggested that gastrin might suppress GC cells by increasing AE2 expression and that gastrin may stimulate AE2 expression in GC cells via early growth response 1 (EGR1) in a cholecystokinin B receptor (CCKBR)-dependent manner [37], demonstrating that AE2 plays a role in carcinogenesis. Furthermore, Wu et al. reported that ectopic expression of AE2, AE1, and p16 is an important pathogenic factor in the development of GC and that dysfunctional AE2 can be degraded by a ubiquitin-dependent pathway [38]. Destruction of AE2 leads to cell alkalization and gastric acid deficiency [97], while AE1/p16 expression leads to the downregulation of AE2, which aggravates cell alkalization and gastric acid deficiency, both of which are characteristics of GC. Similarly, knockdown of AE1 expression with synthetic small interfering RNA (siRNA) significantly inhibited GC growth and reduced the tumor formation rate in a mouse GC model. In addition, the rate of GC formation at the end of the experiment decreased simultaneously with the incidence of gastric atypical hyperplasia, suggesting that AE1 RNA interference (RNAi) therapy may inhibit the formation of gastric tumors by blocking GC progression [99]. In summary, AE1 may function as a cancer-promoting gene for GC and AE2 plays a role in carcinogenesis, indicating that these proteins are potential targets for the treatment of GC.

## 4. Physiological Characteristics of NHEs in the Stomach and Pathophysiological Relevance in GC

### 4.1. Physiological Function of the NHE Family in the Stomach

The mammalian NHE family has 10 members, and each member has its own cellular localization and tissue distribution. NHEs have broad physiological functions, including pHi homeostasis, cell volume regulation, acid-base regulation, and electroneutral NaCl transport [100, 101]. Multiple NHE isoforms are expressed in the stomach; NHE1, NHE2, and NHE4 are expressed in the stomach and play important roles in gastric cell volume and pHi regulation [102]; thus, we will discuss NHE1, NHE2, and NHE4 in the stomach.

NHE1 is the most direct pH regulator and has become a focus of research in recent years [103]. NHE1 expression and function in the stomach have been demonstrated in healthy humans [104]. This protein determines pHi by catalyzing the electroneutral exchange of extracellular Na^+^ and intracellular H^+^ [105]. Epithelial NHE2 is encoded by the *SLC9A2* gene, has 812 amino acids, and is localized on the basolateral membrane of the stomach [106, 107]. Parietal cells are missing in Slc9a2 knockout mice, consistent with the involvement of NHE2 in preventing or responding to damage [108]. NHE4 is highly expressed in the stomach, where it is localized on the basolateral membrane of parietal cells [100]. Deletion of NHE4 causes morphological changes in the gastric mucosa, including a loss of parietal cells and mature chief cells and an increase in the number of undifferentiated cells, necrotic cells, and apoptotic cells. The researchers concluded that NHE4 functionally couples with AE2 (SLC4A2) to maintain cell volume and intracellular ion concentrations for acid secretion [109]. However, the functions and molecular mechanisms of NHE1, NHE2, and NHE4 in regulating gastric cell volume and pHi are not fully understood.

### 4.2. Pathophysiological Role of NHEs in GC

NHE1 can determine pHi by transporting electroneutral extracellular Na^+^ and intracellular H^+^ [105]. Some experiments have shown that NHE1 expression is higher in GC tissues and cell lines than in normal tissues and cell lines. Loss of NHE1 inhibits GC cell proliferation, migration, and invasion in vitro, and NHE1 inhibition reduces GC tumor growth in nude mice. Moreover, NHE1 regulates these events through changes in the pHi and the expression of corresponding genes, and modulation of NHE1 and its downstream signaling pathways could be a novel therapeutic strategy for human GC [26]. Therefore, NHE1 may be a potential target in the treatment of GC. However, more research should be performed to clarify the pathophysiological functions of NHE2 and NHE4 in GC because of their important roles in the normal stomach.

## 5. Physiological Characteristics of K^+^ Channels in the Stomach and Pathophysiological Relevance in GC

### 5.1. Role of K^+^ Channels in the Stomach

Potassium (K^+^) channels are located in cell membranes and control K^+^ ion efflux and influx [110] to play crucial roles in both excitable and nonexcitable cells. Based on the structure, activation mechanisms, and function, K^+^ channels are classified into four main classes: calcium-activated K^+^ (KCa) channels, voltage-gated K^+^ (Kv) channels, inward-rectifier K^+^ (Kir) channels, and two-pore domain K^+^ (K2P) channels. The Kv channel family can be subdivided into Kv1–4 channels (Shaker-, Shab-, Shaw-, and Shal-like subunits); silent Kv5, Kv6, Kv8, and Kv9 subunits (regulators); Kv7 channels (KCNQ); and Kv10–12 channels (EAG-like) [111, 112]. K^+^ channels influence gastric acid secretion by mediating the pumping in and out of K^+^ and maintaining the K^+^ concentration to help H^+^/K^+^-ATPases pump H^+^ into the lumen. Multiple K^+^ channels have been reported to be involved in gastric acid secretion, and dysfunction of K^+^ channels leads to an imbalance in gastric mucosa ion homeostasis and impaired gastric acid secretion [113–115], which may further promote the development of CAG and GC [27–31].

### 5.2. Pathophysiological Function of K^+^ Channels in GC

Numerous K^+^ channels have been shown to play an important role in the development and progression of GC [27–31]. Deletion of Kir2.2 plays a role in the escape of cancer cells from premature senescence and in suppressing tumorigenesis *in vivo* by increasing the levels of the tumor suppressor p27 and ROS accumulation to inducing cellular senescence [29]. In the Kv channel family, Kv4.1, Kv7.1 (KCNQ1), and Kv1.5 have been identified to be involved in promoting GC cell proliferation and progression [27, 28, 30, 31]. Furthermore, the KCNQ1 subunit KCNE2, which is downregulated in GC, was demonstrated to suppress cell proliferation and tumorigenesis of the stomach [116]. Additionally, although Kv11.1 was not detected in the normal stomach, Kv11.1 expression was upregulated in GC tissues [32], and Kv11.1 has been shown to enhance the proliferation and tumorigenesis of GC both *in vitro* and *in vivo* and modulate vascular endothelial growth factor 1 (VEGF-1) secretion through an AKT-dependent pathway (Figure 2) [117, 118]. Moreover, Kv11.1 has been demonstrated to be necessary for the cisplatin-mediated induction of apoptosis in GC, suggesting that this channel may be a new potential target for cisplatin chemotherapy [119]. Thus, the correction of K^+^ channel disorders may be another effective therapeutic strategy for GC. The molecular mechanism of the dysfunction of multiple ion transporters and channels in GC onset is summarized in Figure 2.

## 6. Conclusion

Multiple ion transporters and channels in normal gastric mucosal epithelial cells regulate gastric acid secretion, ion transport, and fluid absorption and thus play an important role in maintaining acid/base homeostasis. Dysfunction of these ion transporters results in disordered ion transport, mucosa barrier dysfunction, and impaired acid/base homeostasis, leading to the development of gastric acid-related diseases, including CAG and GC. We summarize the specific localization, transport type, and function of ion transporters that are involved in acid secretion and the role their dysfunction plays in GC in Figures 1 and 2 and Table 1. Although the physiological and pathophysiological roles of these ion transporters have been described, basic and genetic research is still required to fully elucidate these functions to provide promising therapeutic targets for CAG and GC. In this review, we provide a basic and systemic description in this field to prompt researchers to focus on the functional diversity of ion transporters and channels in the stomach and their role in GC onset, which will provide a novel perspective not only for GC therapy but also for prevention.

## Figures and Tables

**Table 1:** Expression, localization, and physiological and pathophysiological functions of ion transporters in the normal gastric epithelium and GC.

Ions	Related transporters	Human gene symbol	Parietal localization	Transporter type	Physiological role in gastric acid secretion	Pathophysiological relevance in GC
Cl^−^	CFTR	ABCC7	Apical	O	Pumps Cl^−^ out of parietal cells to form HCl with H^+^	CFTR expression is upregulated in GC [23] and is closely related to CA199 [24].
	CLC-2	CLCN2	Apical	O	Pumps Cl^−^ out of parietal cells to form HCl with H^+^	Loss of CLC-2 influences acid secretion and causes precancerous changes [25].
	CLIC-6	CLIC6	Apical	O	Pumps Cl^−^ out of parietal cells to form HCl with H^+^	

H^+^	NHE1	SLC9A1	Basolateral	E	Na^+^-H^+^ exchanger pumps out redundant H^+^ and pumps in Na^+^ at the basolateral side	NHE1 expression is upregulated in GC, and functional data show that loss of NHE1 inhibits GC cell proliferation, migration, and invasion [26].
	NHE2	SLC9A2	Basolateral	E		
	NHE4	SLC9A4	Basolateral	E		

K^+^	KCNQ1	KCNQ1	Apical	O	Pumps K^+^ into the lumen	KCNQ1 is implicated in GC progression [27, 28].
	Kir2.2/4.1/5.1	KIR	Apical	O	Pumps K^+^ into the lumen	Kir2.2 plays a role in the escape of cancer cells from premature senescence and in tumor formation [29].
	Kv1.5/4.1/7.1/11.1	KCNA/D/Q/H	Apical	O	Pumps K^+^ into the lumen	Kv1.5/4.1/7.1/11.1 promotes GC cell proliferation and progression [27, 28, 30–32].
	NKCC1	SLC12A2	Basolateral	C	Na^+^-K^+^-2Cl^−^ cotransporter pumps Na^+^, K^+^, and 2Cl^−^ into parietal cells	

HCO_3_^−^	AE1	SLC4A1	Basolateral	E	Cl^−^-HCO_3_^−^ exchanger pumps Cl^−^ into and HCO_3_^−^ out of parietal cells	AE1 may function as an oncogene in GC [33].
	AE2	SLC4A2	Basolateral	E	Cl^−^-HCO_3_^−^ exchanger pumps Cl^−^ into and HCO_3_^−^ out of parietal cells	The cytoplasmic AE1/p16 complex plays a key role in GC progression [33–35].
	SLC26A7	SLC26A7	Basolateral	E	Cl^−^-HCO_3_^−^ exchanger pumps Cl^−^ into and HCO_3_^−^ out of parietal cells	AE2 may play a role in carcinogenesis [36–38].
	NBCe1	SLC4A4	Basolateral	C	Na^+^-HCO_3_^−^ cotransporter pumps Na^+^ and HCO_3_^−^ out of parietal cells	

C: cotransporter; E: exchanger; O: orphan transporter.
